# Summering on the bank: Seasonal distribution and abundance of monkfish on Georges Bank

**DOI:** 10.1371/journal.pone.0206829

**Published:** 2018-11-05

**Authors:** Liese A. Siemann, Carl J. Huntsberger, Jasper S. Leavitt, Ronald J. Smolowitz

**Affiliations:** 1 Coonamessett Farm Foundation, Inc., East Falmouth, Massachusetts, United States of America; 2 Darling Marine Center, University of Maine, Walpole, Maine, United States of America; 3 Department of Biology, Trent University, Peterborough, ON, Canada; Cornell University, UNITED STATES

## Abstract

The American monkfish is an important commercial species that is widely distributed across a range of depths and temperatures from North Carolina to southern Nova Scotia, including on Georges Bank. We examined changes in the seasonal distribution and relative abundance of monkfish in the scallop access areas in Closed Area I and Closed Area II on Georges Bank using catch data from a three-year seasonal scallop dredge survey. Over the course of the survey, more than 6,000 monkfish were caught and measured, and clear seasonal changes in monkfish abundance were documented. Monkfish catch peaked in the summer and early fall when they were caught across the entire survey area, while they were caught only in deeper waters at the edges of the bank in the winter. Monkfish relative abundance was modeled using a generalized linear mixed model with a Tweedie distribution, and the final model, with month, depth, and bottom temperature as fixed effects, effectively explained the seasonal shifts in the location and relative abundance of monkfish observed during this study. The results suggest that monkfish movements are driven by seasonal changes in bottom temperature. Management measures for monkfish are determined primarily based on data collected during the Northeast Fisheries Science Center bottom trawl surveys, yet this survey catches few monkfish, adding uncertainty to stock assessments. Our research indicates that increasing the use of dredge surveys to collect data on monkfish would be a positive step toward improving monkfish assessments. If monkfish movements are impacted by changes in thermal habitat, their distributions may shift in response to climate change, increasing the need for improved monkfish assessment strategies to effectively manage the species in the future.

## Introduction

The American monkfish (or goosefish, *Lophius americanus*) is an important commercial species that is common along the continental shelf and slope from North Carolina to southern Nova Scotia [1–4]. Monkfish are widely distributed across a range of depths (inshore to >900 meters) and temperatures (0°to 24°C), and they are caught in all sampled depths and temperatures during the Northeast Fisheries Science Center (NEFSC) annual seasonal surveys [3,5]. Early genetic analysis indicated that the American monkfish population was a single genetic stock, with no differentiation between individuals caught from the North Carolina to Maine [6], but more recent studies revealed genetic differentiation between American monkfish caught north and south of Delaware Bay where its range overlaps that of the congeneric blackfin goosefish (*Lophius gastrophysus*) [7–8].

**Fig 1:**
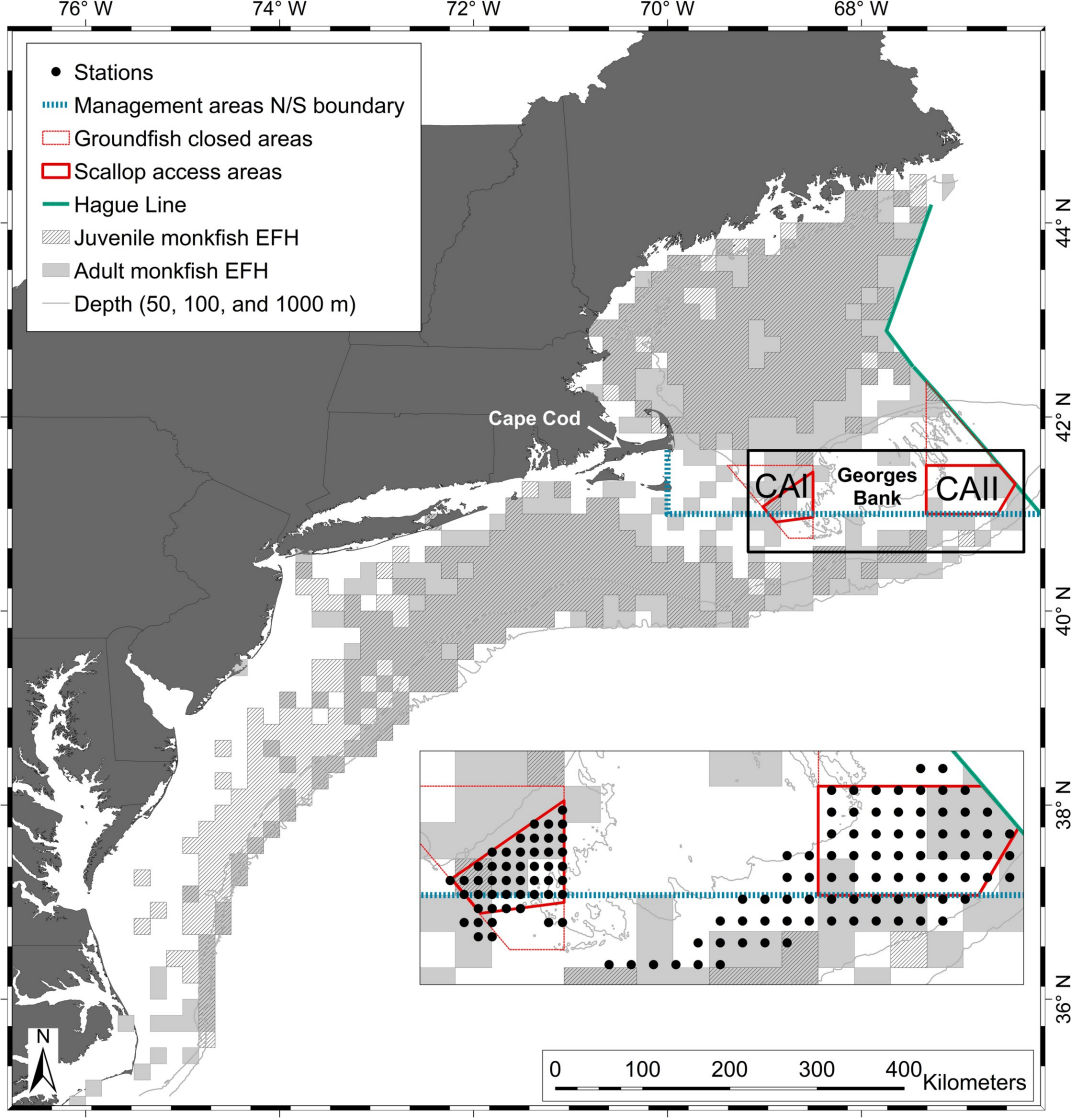
Location of bycatch survey project and stations. The bycatch survey stations (inset) covered scallop access areas in Closed Area I and Closed Area II. The designated boundaries of current Essential Fish Habitats for adult (light gray) and juvenile (hatched) monkfish are also shown (GIS shapefiles from http://www.nmfs.noaa.gov/sfa/hms/documents/fmp/am1/shapefiles.html).

The monkfish Fishery Management Plan (FMP) was created in response to significant increases in monkfish landings in the mid- to late- 1980s, and it first took effect in 1999 [9–10]. Monkfish are currently managed as two units, with the boundary between the Northern Fishery Management Area (NFMA) and Southern Fishery Management Area (SFMA) cutting across Georges Bank at 41°N latitude (**Fig 1**). Although there is no evidence of distinct northern and southern biological stocks corresponding to this boundary [3,9], recent tagging studies indicate that there is limited movement between management areas, with all observed movements occurring from the NFMA to the SFMA [11–12]. The fishery is managed through a days-at-sea system coupled with trip limits, and fishing is permitted year round. Landings peak in spring in both management areas [13]. Most commercial catch is taken by trawl gear in the NFMA, in a fishery that is integrated with the Northeast multispecies fishery [13]. In the SFMA, monkfish are caught mainly in a targeted gillnet fishery [13]. Vessels with commercial scalloping permits can land monkfish, and scallop dredges accounted for up to 40% of landings in the NFMA and half of landings in the SFMA in the 1980s and 1990s [3,9,13]. In recent years scallop vessels have not targeted monkfish due to high scallop catches.

Monkfish seasonal inshore-offshore migrations are regularly reported in the literature [2–3] and in management documents [4–5,14]. Yet seasonal changes in monkfish abundance indices have suggested seasonal movements in opposite directions. In the early 1900s, Tracy (1906) stated that monkfish were common in inshore Rhode Island waters from April until July and in October, but absent during summer months [15]. He hypothesized that monkfish migrated into deep waters during the summer to avoid warm temperatures because monkfish were caught in depths of 240–730 meters in September, while they were distributed widely in shallower waters in the spring and fall. Yet recent surveys indicate that more monkfish inhabit shallow waters during summer months and move into deep waters during the winter, a trend opposite from what was documented previously [3,16].

Tagging studies offer a more direct method for tracking migrations. A single monkfish was caught and tagged on the northern edge of Georges Bank in December 2003 and caught six months later near Cape Cod, Massachusetts [17]. More recently, a total of 607 data storage tags were implanted in monkfish off the eastern coast of the US from offshore Gulf of Maine to Virginia between 2008 and 2014, and 46 of these fish were recaptured [11–12]. Inshore migrations were documented for four monkfish tagged off the continental shelf in Block Canyon in April 2011. Each fish moved into shallow inshore southern New England waters by late May, and these inshore migrations moved the monkfish into warmer waters during with temperatures increasing from lows of 4–6°C to highs of 20–25°C; however, one fish stayed inshore through February of the following year as temperatures approached 0°C [12]. While these studies showed that some monkfish migrate between deeper offshore waters in the winter and shallow inshore waters in the summer, they did not differentiate between movements toward shore versus movements into shallower depths or preferred temperatures due to limited sample sizes, release dates, and locations.

The NEFSC spring (March–May) and fall (September–November) bottom trawl survey is the main source of data used to define Biological Reference Points for monkfish stock assessments and Essential Fish Habitats for juvenile and adult monkfish [3,5,10,14,18–19]. These seasonal surveys, conducted twice a year along the east coast of the United States out to depths of >350 meters, use a lined survey trawl modeled after commercial fishing gear [3]. Additional surveys using shrimp survey and commercial trawls and scallop survey dredges have been used to supplement the data collected by the NEFSC spring and fall surveys when possible [3,5]. Because none of these surveys provide sufficient data at an appropriate scale throughout the year for examining seasonal changes in monkfish distributions on Georges Bank, we analyzed catch and environmental data (depth and temperature) collected during a three-year seasonal scallop dredge survey focused on the scallop access areas in Closed Area I (CAI) and Closed Area II (CAII) (**Fig 1**). While this survey covered a much smaller area than the NEFSC seasonal trawl surveys, the higher spatial and temporal resolution provided an opportunity to examine the movements and distribution of monkfish in a new way.

The primary objective of this study was to describe and model seasonal changes in monkfish distribution and relative abundance on Georges Bank. In addition, scallop dredges were assessed as monkfish survey gear by comparing catch per unit area and length frequencies from this study with catch statistics from the NEFSC bottom trawl survey. Finally, we examined the shifts in monkfish catch relative to depth and temperature, and because the shallow stations were located in the center of the survey area rather than closer to shore, investigated if seasonal movements were between inshore and offshore areas or shallow and deep waters.

## Materials and methods

### Seasonal bycatch survey

All fishing surveys were approved by the National Marine Fisheries Service through Exempted Fishing Permits issued by the Northeast Regional Administrator to the Coonamessett Farm Foundation and the fishing vessels participating in the research.

We conducted 27 survey trips aboard commercial sea scallop fishing vessels between May 2011 and March 2014. Surveys were performed monthly from May 2011 until November 2011, and then every six weeks from January 2012 until March 2014. The sampling locations were located in CAI and CAII on Georges Bank, with some additional stations south and west of CAII (**Fig 1**). The survey used a fixed grid design, with 31 stations sampled during every trip in CAI and 44 stations sampled during every trip in CAII, with 16 additional stations sampled in the open areas adjoining CAII during the final year of the study. Station depths ranged from 37 to 108 meters. CAI stations were separated by 5.4 km east to west and 7.2 km north to south, while CAII stations were separated by 8.6 km east to west and 11.1 km north to south. Open area stations (areas open for scallop fishing year round) added in 2013 were grouped with CAII stations for all analyses.

During each survey, two commercially rigged scallop dredges were towed from the vessel: a standardized 4.57m-wide turtle-deflector dredge (TDD) (**Table 1**) and a New Bedford-style dredge (NBD) supplied by the vessel or by Coonamessett Farm Foundation (CFF) [20]. Each tow passed through the center of the pre-determined grid cell from a start point decided by the vessel captain. Under ideal conditions, the tows were 30 minutes long at 4.8 knots (8.89 km/h). If a tow lasted less than twenty minutes or had gear complications, it was declared invalid and the station would be towed a second time. Start coordinates, depth, and temperature were recorded for each tow, with temperature and depth recorded every 30 seconds by two loggers (Vemco Minilog temperature logger and Star-Oddi DST milli-TD temperature-depth logger) attached to the dredges.

**Table 1:** Gear specifications for the turtle-deflector dredge.

Specification	Details
Rings	Commercial 4-inch
Width	4.57 meters
Apron	8-by-40 ring
Bag	10-by-40 ring
Sides	6-by-18 ring
Skirt	2 ring
Twine top	10.5 inch stretched mesh
Hanging ratio	2:1
Turtle mat	present
Ticklers	9 rows
Scope	3:1 + 10

After each tow, scallops and commercially important bycatch species were sorted, counted, and measured. Monkfish were measured to the nearest centimeter from the tip of the nose to the tip of the tail, and then released.

### Data analysis

#### Mapping seasonal changes in monkfish abundance

Because the NBD was not the same for all trips, analysis of monkfish relative abundance included only catch from the TDD (referred to as the CFF dredge). Averages per trip of monkfish counts and the number of stations with monkfish present were plotted over time to examine changes in both of these summary statistics. To examine how total, juvenile, and adult monkfish abundance was distributed relative to sampled bottom temperatures and depths, the unweighted and abundance-weighted cumulative frequency distributions of each variable were plotted by season, and the cumulative distributions were compared using a two-sample Kolmogorov-Smirnov test. Using the tow midpoints, based on coordinates recorded at sea, to specify survey locations, the abundance of monkfish caught in the CFF dredge was plotted by month using ArcGIS. The abundance of juvenile and adult monkfish were also plotted by season (winter: December-February, spring: March-May, summer: June-August, fall: September-November), with monkfish categorized based on the 50% length at maturity cutoff of 43 cm used for monkfish management [21–22].

#### Comparison of CFF dredge and NEFSC bottom trawl monkfish catch

The NEFSC has conducted seasonal bottom trawl surveys along the northeastern coast of the United States since 1963 using standardized trawls modeled after commercial gear [23]. Although the vessel and gear used for these surveys changed in 2008, the trawl has consistently included a small-mesh cod-end liner to retain small fish [23]. To assess differences between data from the CFF bycatch and the 2009–2014 NEFSC trawl surveys that are used to for monkfish management, we plotted the size frequency data from the seasonal bycatch survey and the NEFSC spring and fall bottom trawl surveys conducted in the areas covered by the bycatch survey (**Fig 1**) (NEFSC trawl survey data, unpublished). The NEFSC trawl survey data used in this comparison was selected by plotting trawl station coordinates in ArcGIS and selecting stations that fell within the boundaries of the bycatch study.

To generally assess trends between monkfish catch in the trawl and dredge, we identified pairs of CFF dredge and NEFSC trawl stations that were spatially and temporally close (depth ≤ 7 meters apart, distance ≤ 6.5 km apart, sampling date ≤ 7.5 days apart). Catch numbers were adjusted for swept area based on the average swept area for the bycatch survey tows and the reported global mean swept area for trawl survey tows [24]. In addition to comparing the adjusted catch numbers, we analyzed changes in the catch ratio (trawl/dredge) at each station pair with the average station depth and bottom temperature for all pairs with catches greater than zero for one gear type.

#### Modelling seasonal changes in monkfish distributions

The number of monkfish per tow was modeled in the R package “mgcv” (generalized additive model function “gam” with family = “Tweedie” and link = “log”) [25–26]. We used a model with a Tweedie distribution because the count data was over dispersed with a high proportion of zero values [27–28]. Fixed effects available for modeling the monkfish catch numbers included location (“easting” and “northing” with latitude and longitude coordinates projected into UTM space using the R package “rgdal”) [29], bottom depth (in meters), bottom temperature (rounded to the nearest °C), area (CAI or CAII), and month (as categorical variables). Prior to running the mixed models, we looked for evidence of high collinearity between the fixed effect variables using variance inflation factors (VIFs) and examined the data for evidence of spatial auto-correlation with the spline correlogram function “spline.correlog” available in the R package “ncf” [30–31]. Scatter plots of location, depth, and bottom temperature versus monkfish catch by month were examined to determine if quadratic terms should be included.

Random effects for survey trip and survey station were added to the models to account for differences in monkfish numbers due to changes in survey vessels or survey protocol and any consistent differences between survey stations that might affect dredge operation. The final model was determined based on the Akaike Information Criterion (AIC) after backward elimination of variables and interaction terms [32]. Models were evaluated using AIC weights from the function “akaike.weights” in R package “qpcR” [33]. The significance of the fixed effects in the final model were assessed using the function "anova.gam" in the R package "mgcv", a function that uses a Type III ANOVA to assess model terms [26]. To visualize model fit, the observed catch numbers per tow were averaged by station and used to create smoothed kernel density plots for each season with the spatial analyst tools in ArcGIS. The fitted values from the final model were sorted by season and plotted over the kernel density plots.

## Results and discussion

**Fig 2:**
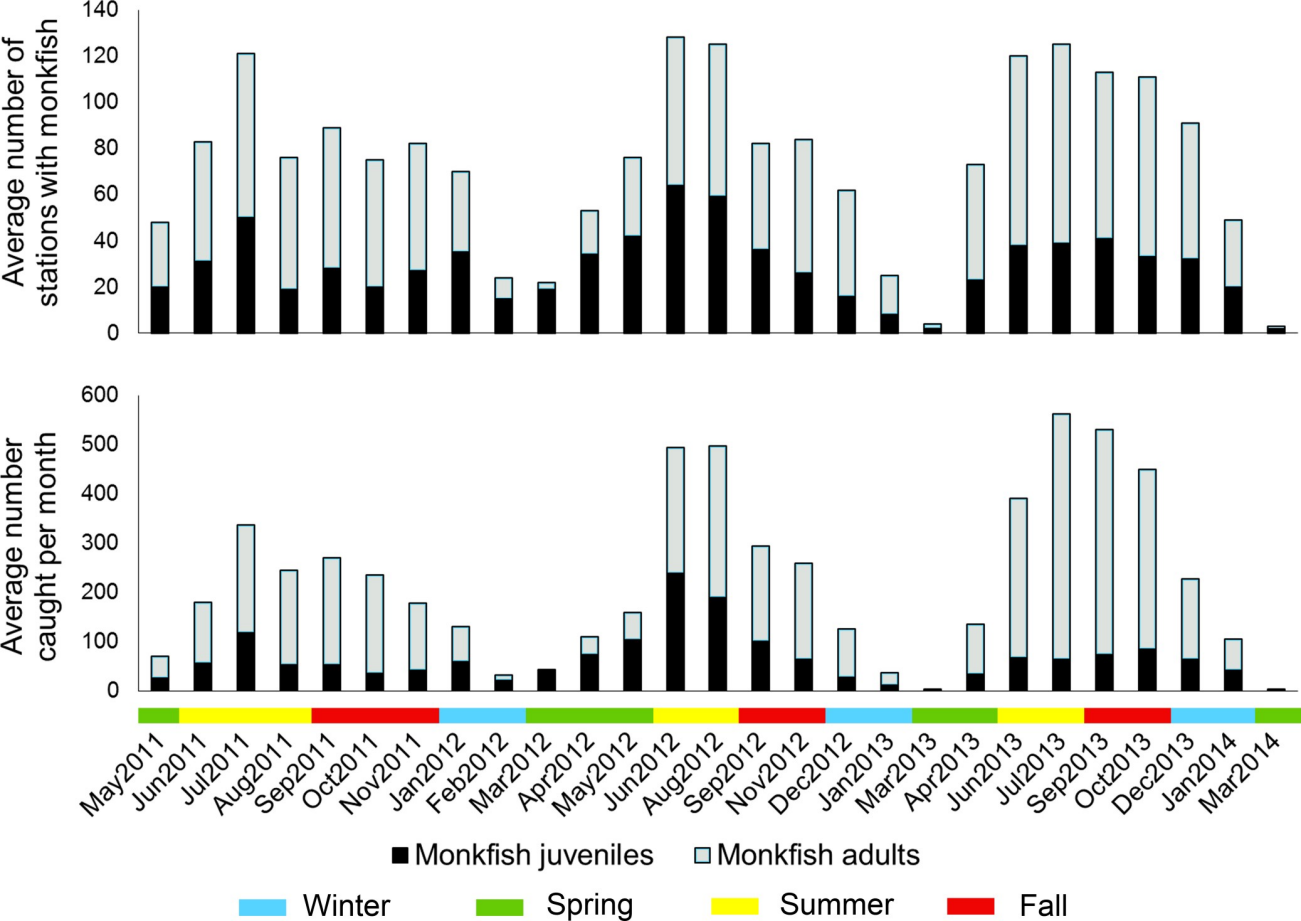
The average number of stations with monkfish and the average number of monkfish caught per trip by month from May 2011 to March 2014. Monkfish juveniles are less than 43 cm in length.

Over the course of the three-year dredge survey, 6,117 monkfish were caught in both closed areas, with 1,999 caught in CAI and 4,118 caught in CAII and the open area to the southwest (**Table 2**). Catches peaked in July in CAI and September in CAII (**Table 2**). Overall, 71% of the monkfish were adults (size > 43 cm long), although this percentage decreased to less than half of the catch in February, April, and May in CAI and February, March, and May in CAII (**Table 2**). Plots of the average number of stations with monkfish present (**Fig 2**) and average monkfish counts (**Fig 2**) over the course of the survey show clear seasonal changes, with monkfish presence and catch numbers in CAI and CAII peaking in the summer and early fall.

**Table 2:** Average monkfish catch per month and total catch for Closed Area I, Closed Area II, and overall.

		Closed Area I		Closed Area II		Overall	
Month	Number of years sampled	Average catch per month	% Adult (>43 cm)	Average catch per trip	% Adult (>43 cm)	Average catch per trip	% Adult (>43 cm)
**January**	3	12	62%	79	58%	91	59%
**February**	1	5	40%	28	36%	33	36%
**March**	3	3	50%	14	5%	17	12%
**April**	2	8	44%	115	57%	123	56%
**May**	2	34	31%	82	48%	115	43%
**June**	3	167	60%	188	71%	355	66%
**July**	2	199	71%	252	86%	451	80%
**August**	2	145	61%	228	71%	372	67%
**September**	3	91	77%	274	80%	365	79%
**October**	2	103	92%	240	79%	343	83%
**November**	2	71	83%	149	72%	219	76%
**December**	2	29	89%	149	71%	177	74%
**Total catch**		1999	69%	4118	72%	6117	71%

The percentage of the catch that was adults (>43 cm long) is also shown for each month and overall.

**Fig 3:**
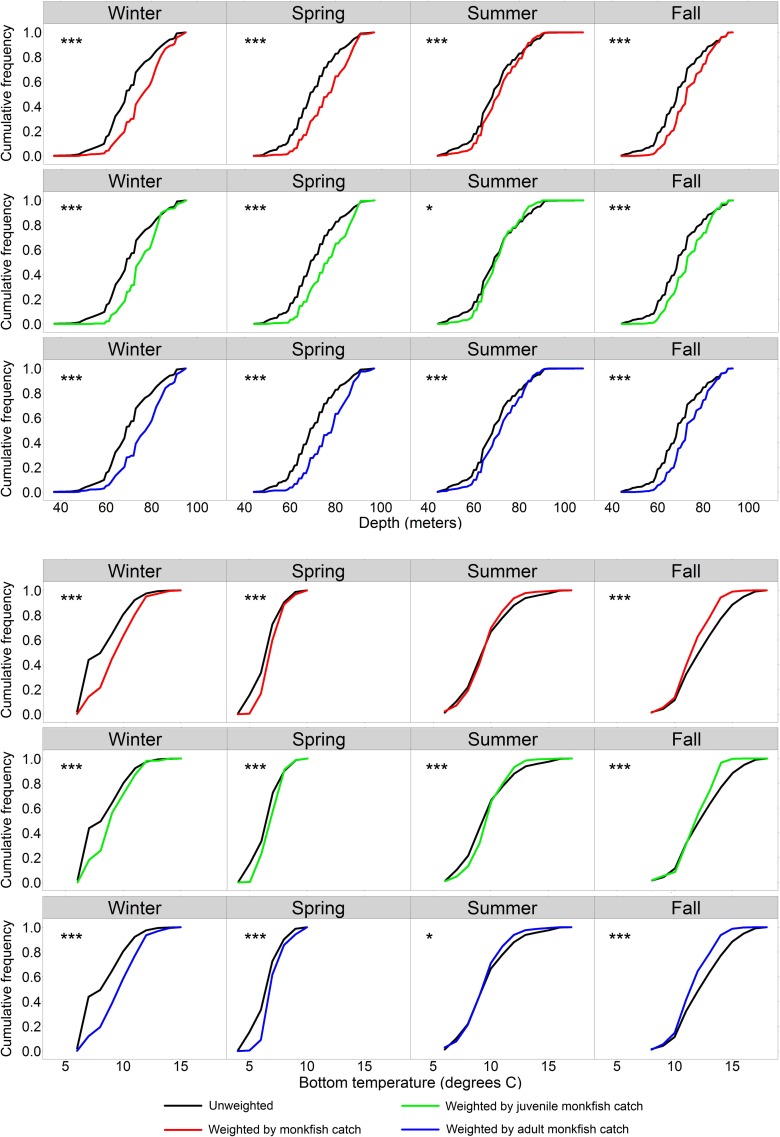
Unweighted (black) and abundance weighted cumulative frequency distributions (red: Total catch, green: Juvenile catch, and blue: Adult catch) of sampling-station bottom depths and temperatures for each season. Asterisks indicate significant shifts in the abundance-weighted curves relative to sampled depths and temperatures (two-sample Kolmogorov-Smirnov test, * *p* < 0.05 and *** *p* < 0.001).

During all seasons, total and adult monkfish catch distributions at depth were shifted toward deeper waters relative to sampled depths (two-sample Kolmogorov-Smirnov test, *p* < 0.001) (**Fig 3**). Juvenile monkfish were also caught in relatively deeper waters in the winter, spring, and fall (two-sample Kolmogorov-Smirnov test, *p* < 0.001), but their distribution was contracted toward middle depths in the summer (two-sample Kolmogorov-Smirnov test, *p* < 0.05) (**Fig 3**). Monkfish were rarely caught at the shallowest stations (30–50 m) during most of the year.

During the summer, monkfish catch distributions were narrowed toward middle of sampled bottom temperatures, with this shift being statistically significant for juvenile (two-sample Kolmogorov-Smirnov test, *p* < 0.001) and adult monkfish (two-sample Kolmogorov-Smirnov test, *p* < 0.05). The abundance weighted cumulative frequency curves for bottom temperature were shifted during the winter, spring, and fall (two-sample Kolmogorov-Smirnov test, *p* < 0.001) (**Fig 3**). In the winter and spring, monkfish catch (total, juvenile, and adult) was higher in warmer waters when bottom water temperatures overall were low. The opposite trend was observed in the fall, with monkfish catch peaking in relatively colder waters.

**Fig 4:**
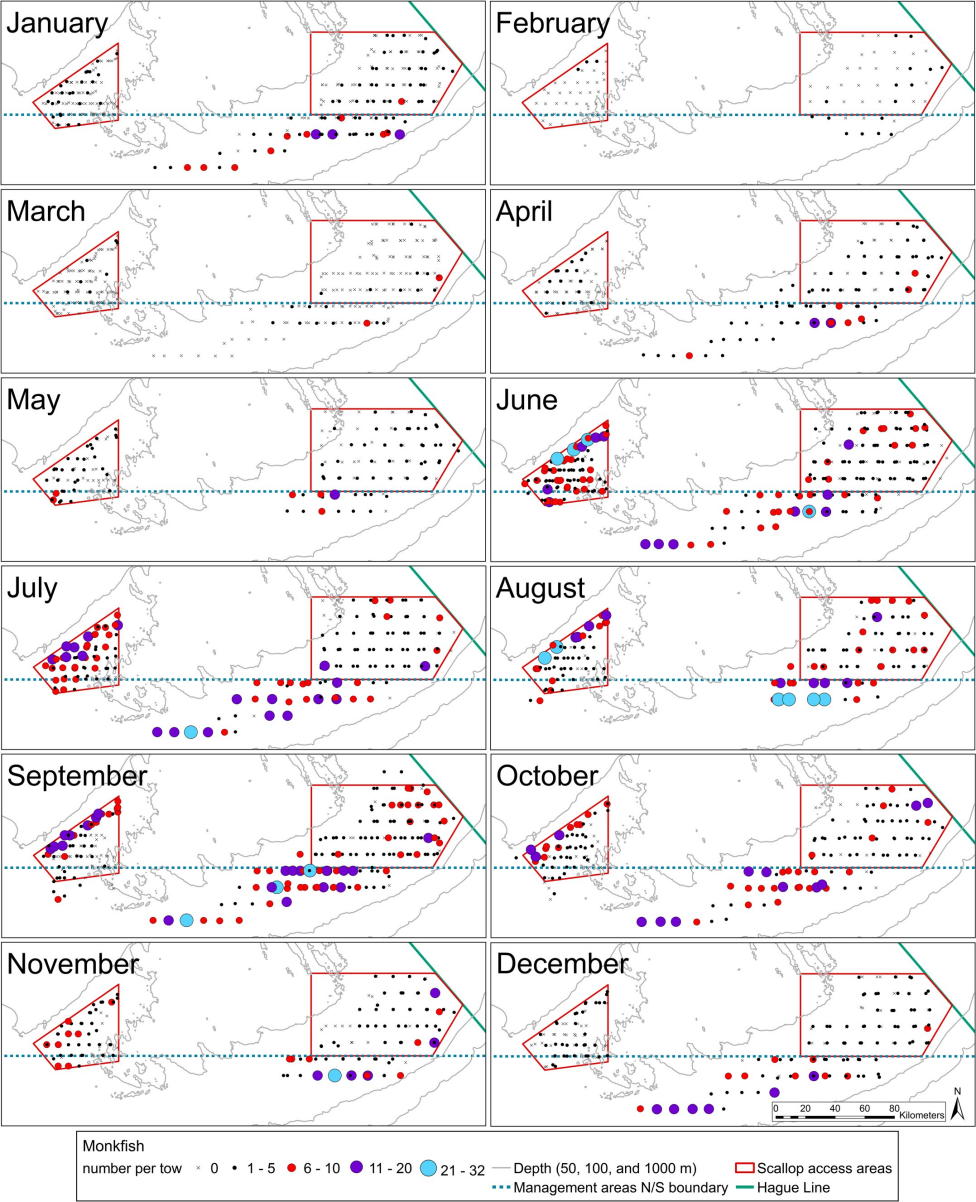
Maps showing the number of monkfish caught per station during each month. Over the course of the three-year survey, stations were sampled three times in January, March, June, and September; two times in April, May, July, August, October, November, and December; and once in February.

**Fig 5:**
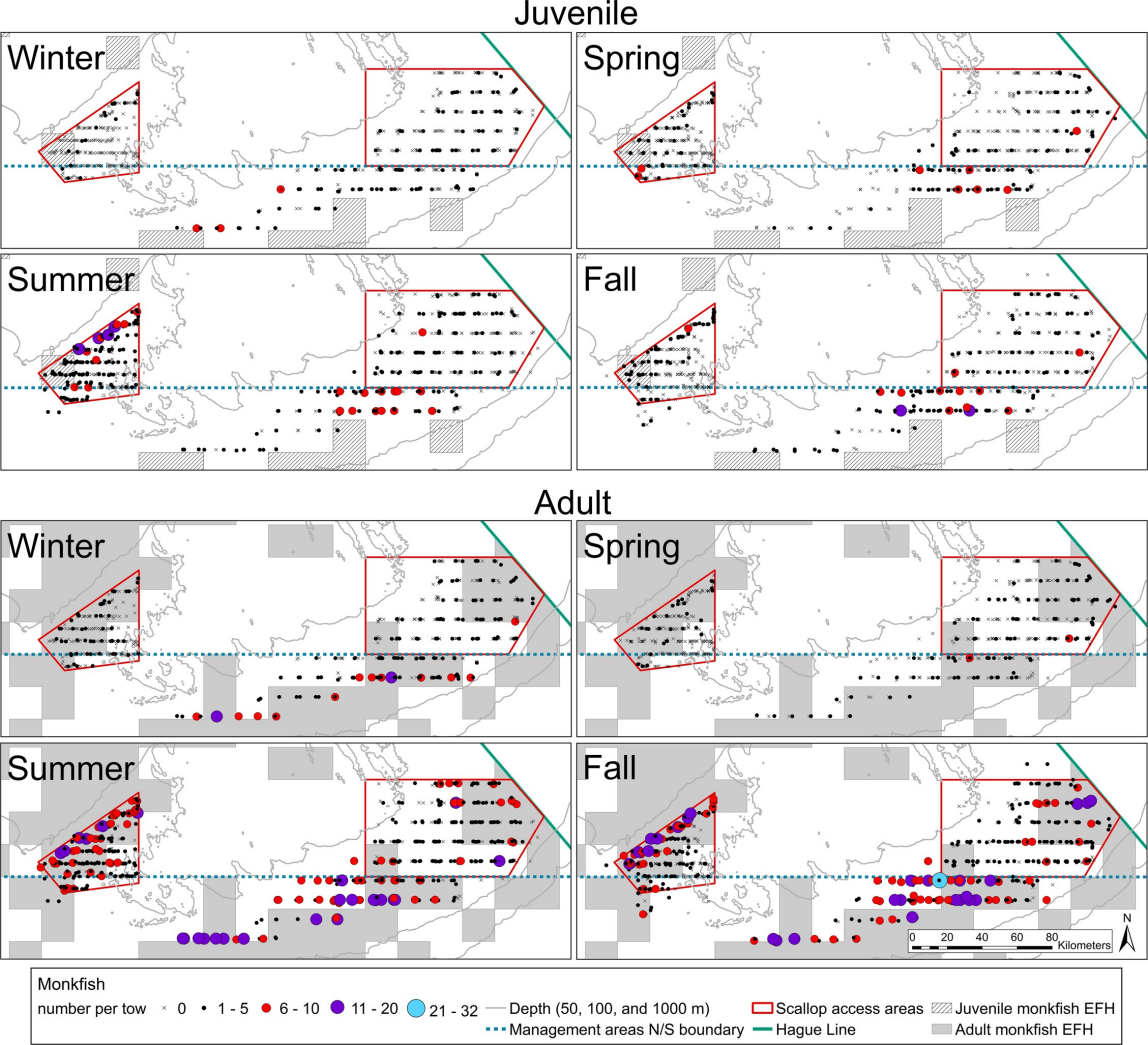
Maps showing the number of juvenile and adult monkfish caught per station during each season (winter: December-February, spring: March-May, summer: June-August, fall: September-November). Station catch data is overlaid on the Essential Fish Habitat locations for each group.

Throughout the summer and fall months, the largest monkfish catches occurred in deeper waters near the edge of the Georges Bank (**Figs 4** and **5**). In June and July, monkfish were caught across most of CAI, while during August through October, the larger monkfish catches (> 5 fish) occurred along the northern edge of CAI at depths of 70–100 meters (**Fig 4**). The largest catches on eastern Georges Bank took place in August and September, primarily south of CAII at depths of 70–100 meters (**Fig 4**). Catches decreased throughout the winter, reaching the lowest levels in February in both areas, when no monkfish were caught at most stations (**Fig 4**). Larger catches of adult monkfish (> 10 fish) occurred in the summer and fall in both closed areas at depths of 70–100 meters, while similar catches of juvenile monkfish were limited to the northern edge of CAI in the summer and south of CAII in the fall at depths of 70–90 meters (**Fig 5**).

### CFF dredge vs NEFSC bottom trawl surveys

This analysis included 96 spring tows and 124 fall tows from the NEFSC surveys conducted from 2009 to 2014. The CFF bycatch study data set included 491 tows in the winter, 522 tows in the spring, 523 tows in the summer, and 524 tows in the fall.

**Fig 6:**
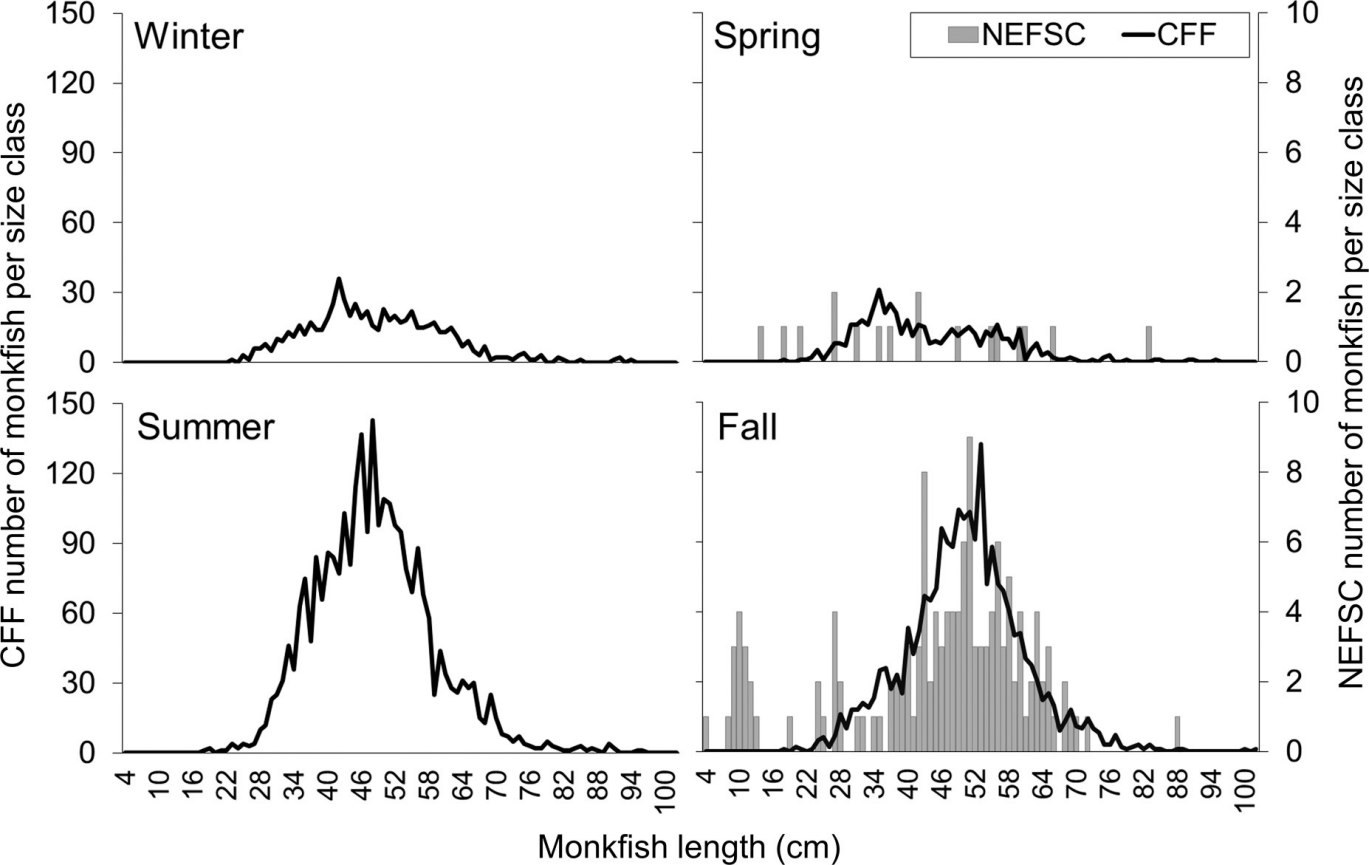
Number of monkfish caught per size class by season during the bycatch survey from May 2011-March 2014 (black lines) and the spring and fall NEFSC bottom trawl surveys from spring 2009-fall 2014 in the same area (gray bars).

The largest monkfish catch with the dredge occurred during the summer months of June to August (2,711 fish), and the average length of the fish was 48 cm (**Table 3** and **Fig 6**). Monkfish catch with the dredge was also high in the fall from September through November (2,219 fish), with an average monkfish length of 50 cm (**Table 3** and **Fig 6**). Dredge catch was lowest in spring months from March through May (526 fish), with the monkfish catch size frequency shifting toward smaller fish (average monkfish length = 44 cm) (**Table 3** and **Fig 6)**. Monkfish catch with the dredge was also low in the winter months of November through January (661 fish), and the average length of the fish was 48 cm during these months.

**Table 3:** Summary of monkfish catch by season for the CFF seasonal bycatch dredge survey and the NEFSC bottom trawl surveys on the Bigelow limited to the area covered by the bycatch survey.

	**CFF dredge survey (2011–2014)**				
**Season**	**Number of stations**	**Monkfish catch**		**Monkfish average length in cm (range)**	**Monkfish average catch per station (range, 90**^**th**^ **percentile)**
Winter	491	661		48 (23–94)	1.4 (0–16, 4)
Spring	561	526		44 (18–95)	0.9 (0–13, 3)
Summer	567	2,711		48 (18–96)	4.8 (0–26, 10)
Fall	567	2,219		50 (18–102)	3.9 (0–32, 10)
	**NEFSC trawl survey (2009–2014)**				
**Season**	**Number of stations**	**Monkfish catch**	**Swept-area adjusted monkfish catch**	**Monkfish average length in cm (range)**	**Monkfish average catch per station (range, 90**^**th**^ **percentile)**
Spring (CFF area)	96	17	9	43 (11–83)	0.2 (0–2 1)
Fall (CFF area)	124	138	73	44 (4–88)	1.1 (0–13, 3)
Spring full range	2,128	4,290	2,264	42 (6–115)	2.0 (0–108, 6)
Fall full range	2,121	4,941	2,607	33 (4–101)	2.3 (0–53, 8)

Catch in the NEFSC survey trawl was adjusted by swept area to be comparable to catch in the CFF scallop dredge.

Monkfish catch from the NEFSC bottom trawl surveys are also shown in **Table 3** and **Fig 6**. Monkfish catch in the bycatch survey area during fall surveys from 2009 through 2014 was 138 fish, with an average monkfish length of 48 cm. Unlike the dredge catch, the monkfish catch in the bottom trawl included a high proportion of small monkfish in the 4–15 cm range, with 58% of these small fish caught in 2011 (**Fig 6**). Monkfish were rarely caught in the same areas during the spring, with only 17 fish caught at 96 survey stations from 2009–2014 (**Table 3** and **Fig 6)**.

There were 21 pairs of CFF dredge and NEFSC trawl stations that were spatially and temporally close. Station depths and bottom temperatures measured by each survey were within 7 meters and 0.9°C of each other. Depths ranged from 66–82 meters, and bottom temperatures ranged from 6–15°C. Monkfish were caught at 13 of these station pairs. The dredge caught monkfish when the trawl did not at six of these 13 stations, and the dredge always caught more monkfish than the trawl, with an average of seven times more monkfish overall. Despite the small sample size, there was a weak but positive correlation between the number of monkfish caught at the dredge stations and the number caught at the corresponding trawl stations (R = 0.625, R^2^ = 0.390, F(1,12) = 7.047, *p* = 0.022). The catch ratio of the two gear types (trawl/dredge) ranged from 0.125 to 0.5, and it changed with bottom temperature but not with depth. There was a significant positive correlation between the catch ratio and bottom temperatures ranging from 6–15°C (R = 0.762, R^2^ = 0.580, F(1,12) = 15.189, *p* = 0.002).

### Model results

We did not include a spatial variable because there was no strong spatial autocorrelation between sampling stations based on the monkfish count data or residuals from a simple linear model incorporating all of the fixed effect variables (spatial correlation < 0.1 between neighboring stations, lower for more distant stations). Furthermore, longitude was correlated with depth in both closed areas and with latitude in CAII. Bottom temperature was correlated with month, so bottom temperature was not included as a standalone variable if month was in the model. The VIFs for all other fixed variables indicated low to moderate correlations (VIF < 4). Examination of scatter plots of depth and bottom temperature versus monkfish catch numbers indicated that a quadratic model might be appropriate, so we included squared terms for depth and bottom temperature. Interaction effects were included because monkfish catch numbers varied with temperature and depth during each month. The full model; the final model; the models with each effect, polynomial, or interaction dropped; and models with delta AIC values under 10 are shown in **Table 4**. Results of the ANOVA used to determine the significance of the fixed effect terms in the final model are shown in **Table 5.**

**Table 4:** Summary of the Tweedie model analysis and selection using AIC values.

Monkfish numbers per tow (Tweedie parameter = 1.1)					
FULL: Monkfish ~ (BT×Month) + (BT^2^×Month) + (D×Month) + (D^2^×Month) + Area + Trip + Station					
FINAL: Monkfish ~ (BT×Month) + (BT^2^×Month) + (D×Month) + D^2^ + Trip + Station					
Model fixed effects	Edf	Rel LL	AIC	Δ_AIC_	AIC weights
(BT:M) + (BT2:M) + (D:M) + D2	146.331	1.000	7549.453	0.000	0.492
(BT:M) + (BT2:M) + D+ (D2:M)	145.780	0.865	7549.744	0.291	0.425
(BT:M) + (BT2:M) + (D:M) + D2 + M	151.433	0.042	7555.791	6.338	0.021
(BT:M) + (BT2:M) + (D:M) + (D2:M) + M	163.531	0.036	7556.093	6.641	0.018
(BT:M) + (BT2:M) + (D:M) + D2 + M + A	151.708	0.029	7556.537	7.084	0.014
(BT:M) + (BT2:M) + (D:M) + (D2:M) + M + A	164.090	0.025	7556.865	7.413	0.012
(BT:M) + (BT2:M) + D+ (D2:M) + M	151.357	0.021	7557.137	7.684	0.011
(BT:M) + (BT2:M) + D + (D2:M) + M + A	151.686	0.015	7557.898	8.445	0.007
(BT:M) + (BT2:M) + (D:M) + M + A	160.286	0.000	7568.013	18.561	0.000
(BT:M) + (BT2:M) + (D2:M) + M + A	162.930	0.000	7568.857	19.404	0.000
(BT2:M) + (D:M) + (D2:M) + M + A	156.433	0.000	7600.666	51.213	0.000
(BT:M) + (D:M) + (D2:M) + M + A	157.698	0.000	7607.704	58.251	0.000
(D:M) + (D2:M) + M + A	150.346	0.000	7674.114	124.662	0.000
(BT:M) + (BT2:M) + M + A	158.194	0.000	7737.851	188.399	0.000
BT + BT2 + D + D2 + A	124.201	0.000	7824.188	274.735	0.000

Trip and station were included as random effects. BT = bottom temperature, D = bottom depth, M = month, A = Area, Edf = estimated degrees of freedom, Rel LL = relative log likelihood, AIC = Akaike Information Criteria, Δ_AIC_ = delta AIC.

**Table 5:** Significance of the terms in the final model, assessed using a type III ANOVA.

Fixed effect terms	df	F	*p*-value
D2	1	21.676	3.44E-06
BT:M	12	4.962	3.68E-08
BT2:M	12	5.859	4.34E-10
D:M	12	13.223	< 2.00E-16

BT = bottom temperature, D = bottom depth, M = month, df = degrees of freedom.

**Fig 7:**
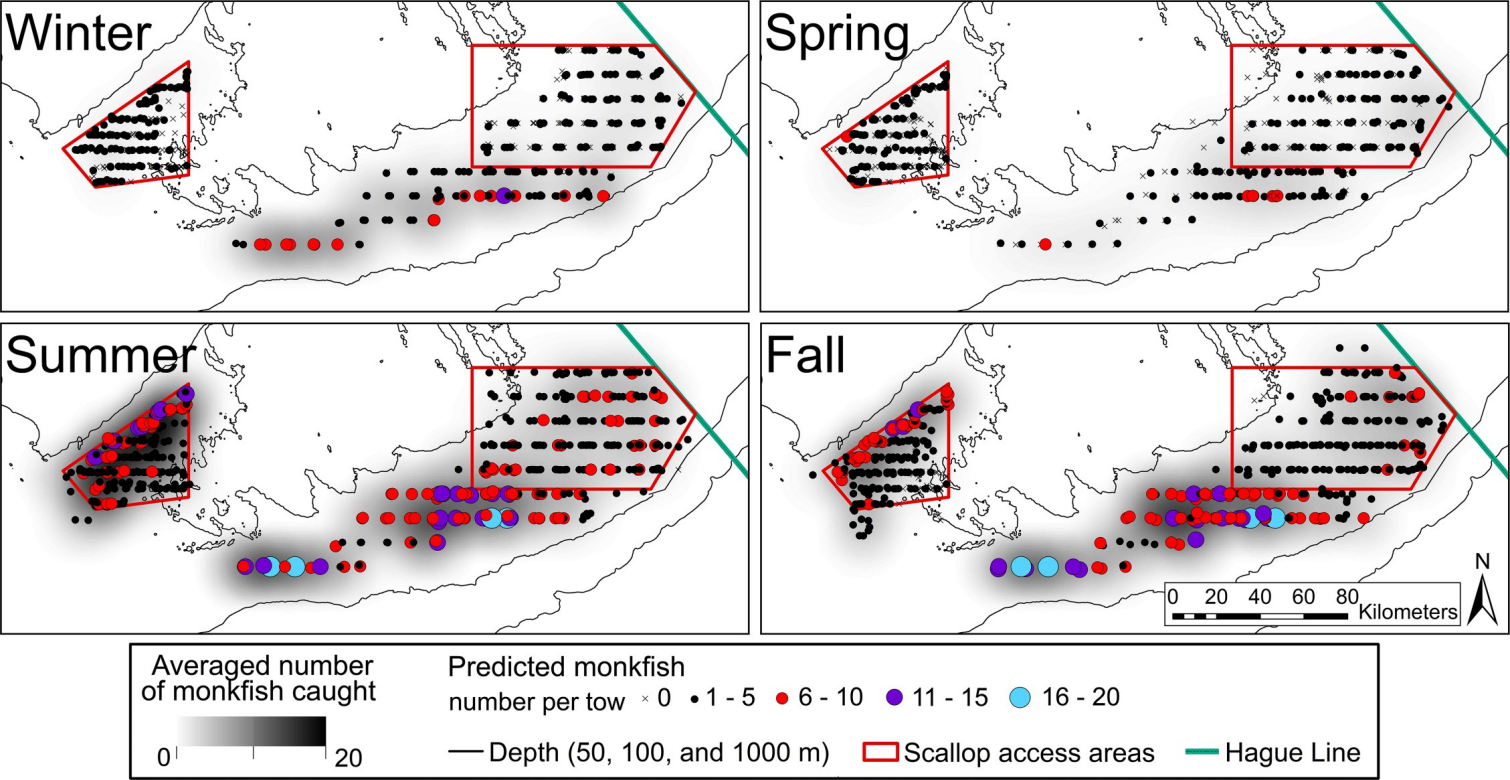
Averaged observed counts for monkfish by season in the bycatch survey study areas (kernel density plots) and predicted monkfish counts from the final Tweedie model.

Based on the AIC weights, the final model for monkfish catch included fixed effects for bottom temperature, depth, and month, and random effects for trip and station. Dropping the fixed effect of survey area and the interaction between depth squared and month resulted in a lower AIC score. Overall, the model accounted for most of the observed variation (deviance explained = 63.7%). The observed and fitted values from the final model were used to create the maps shown in **Fig 7**. Overall, the model correctly predicted the regions where monkfish counts were highest during each season, but the model did not predict the rare high counts of over 20 monkfish per tow.

## Conclusion

During the seasonal bycatch survey, monkfish relative abundance in the closed areas on Georges Bank increased markedly during summer and early fall months. Over the three-year project, there were clear seasonal changes in monkfish relative abundance, with monkfish caught across all of the survey area in the summer but only in deeper waters at the edges of the bank in the winter. Inshore offshore migrations were not apparent. Although monkfish catch was higher at the deeper survey stations throughout the year, this shift was more pronounced during the winter, spring, and fall. The selected model, which incorporated month, depth, and bottom temperature, accounted for the seasonal shifts in the location and relative abundance of monkfish observed during this study.

Bottom temperature and depth are recognized as important determinants of fish distributions [33–41]. Although monkfish are found across a wide range of temperatures, previous studies reported that they are most abundant where bottom temperatures range from 4–14°C [3–4,34]. Our study had similar results, with the majority of monkfish caught when bottom temperatures ranged from 5–14°C. An apparent preference for moderate water temperatures was most obvious during the fall, when sampled bottom temperatures were warmest, and in the winter and spring, when bottom temperatures were cold.

Although monkfish are also found across a wide range of depths, previous research has noted a preference for waters deeper than 70 meters on the Scotian Shelf [16], and relatively deeper than sampled stations during the NEFSC trawl surveys [3–4,41]. We consistently caught more monkfish at stations with depths greater than 60 meters, with over 50% of the catch at stations with depths greater than 70 meters for all months but June and July. The movement of monkfish from shallow waters during summer months into deeper waters during other seasons has been noted for the SFMA and the Scotian Shelf, and both studies suggested that monkfish migrated due to changes in thermal habitat [3,16]. This study also suggests that movements are driven by seasonal changes in bottom temperature. Similar seasonal migrations of Atlantic cod (*Gadus morhua*) have been associated with tracking prey [42–43], and although monkfish are opportunistic feeders [2], prey availability may influence their distributions as well. Because seasonal shifts were observed for both adult and juvenile monkfish, migrations are not clearly linked to reproduction.

The observed catch was greater than 20 monkfish in just under 0.8% of the survey tows, but the final pair of models did not predict these rare extreme values. The values of the predictors for these catches were similar to those observed during the more frequent smaller catches. It is likely that monkfish catch is influenced by variables we could not include in the model. For instance, we recently conducted seasonal dredge surveys and co-located benthic video surveys in Southern New England waters and observed that monkfish catch in scallop dredges was strongly correlated to benthic substrate type (sand, gravel, or rocky substrates), with monkfish caught primarily on sandy substrate (unpublished data). Similar preferences for fine sediments (sand and silt) have been noted previously [3,34].

Previous analysis of monkfish distribution in the Northwest Atlantic, based on NEFSC bottom trawl surveys, concluded that monkfish are not common in shallower waters on Georges Bank [3]. Nevertheless, this survey regularly caught monkfish on Georges Bank at depths of < 50 meters, notably during summer months, with catch numbers per tow that often exceeded NEFSC spring and fall survey catch numbers per tow across the NFMA and the entire survey range. Although the CFF bycatch survey was conducted in the NFMA, the seasonal trend observed in this study agrees more closely with previously reported distribution patterns for monkfish in the SFMA [3]. Richards et al. (2008) summarized monkfish catch data from the NEFSC bottom-trawl surveys in 1948–1949 and 1963–2007. During winter and spring months, monkfish catch numbers were higher at deep stations, while catch was distributed across all station depths during summer months. In fall months, catch was higher in deeper waters in the SFMA, while monkfish were caught across all sampled depths in the NFMA. Seasonal distribution patterns reported for the northern area are effectively those observed in the Gulf of Maine [3], so it is not surprising that monkfish seasonal patterns in CAI and CAII are more similar to those observed in the southern parts of Georges Bank that lie within the SFMA.

Monkfish catchability in the NEFSC survey trawl is low relative to commercial trawl gear and commercial and survey scallop dredges [3]. Our results suggest that uncertainties in the stock assessment that arise from the low bottom trawl catch numbers may be compounded by systematic changes in the relative efficiency of the trawl with bottom temperature. The catch ratio between the two gears is a rough estimate of the relative efficiencies of the two gear types, and the NEFSC trawl/CFF dredge ratio decreased with decreasing bottom temperatures, indicating that monkfish catchability may be particularly low in the bottom trawl relative to the scallop dredge at lower temperatures. Water temperature is known to affect fish swimming speed, herding behavior, and response time to approaching gear [44–46]. Therefore, a change in relative efficiencies is not surprising, but the difference by gear type is puzzling and calls for further investigation to validate this result with a larger sample size.

The need for additional research on all aspects of monkfish biology is widely recognized. The NEFSC seasonal bottom trawl surveys catch few monkfish, adding uncertainty to stock assessments. Furthermore, because the long-term NEFSC seasonal surveys include few stations in each survey strata and they are conducted during only two seasons, important seasonal distribution patterns may be undocumented. Our research indicates that increasing the use of dredge surveys to collect data on monkfish would be a positive step toward improving monkfish assessments. Moreover, current assumptions about a low abundance of monkfish on Georges Bank, as well as the appropriateness of including Georges Bank monkfish in a management unit dominated by Gulf of Maine fish, may need to be reevaluated. If monkfish movements are driven by changes in bottom temperatures, their distributions may shift in response to climate change, increasing the need for improved monkfish assessment strategies to effectively manage the species in the future.
